# *Aurora-A* V57I (rs1047972) Polymorphism and Cancer Susceptibility: A Meta-Analysis Involving 27,269 Subjects

**DOI:** 10.1371/journal.pone.0090328

**Published:** 2014-03-05

**Authors:** Weifeng Tang, Hao Qiu, Heping Jiang, Lixin Wang, Bin Sun, Haiyong Gu

**Affiliations:** 1 Department of Cardiothoracic Surgery, Affiliated People's Hospital of Jiangsu University, Zhenjiang, Jiangsu Province, China; 2 Department of Microbiology and Immunology, Medical School of Southeast University, Nanjing, Jiangsu Province, China; 3 Emergency Department, Affiliated Jintan People's Hospital of Jiangsu University, Jintan, Jiangsu Province, China; Ohio State University Medical Center, United States of America

## Abstract

**Background:**

The association between *Aurora-A* V57I (rs1047972, G>A) polymorphism and cancer susceptibility has been widely studied. However, the results are inconsistent.

**Methodology/Principal Findings:**

To obtain a more precise evaluation of the relationship, we performed a meta-analysis of 14 case-control studies involving a total of 11,245 cancer cases and 16,024 controls. Our results demonstrated that there was a borderline evidence of an association between the *Aurora-A* V57I polymorphism and the decreased risk of overall cancer in two genetic models: AA vs. GA+GG and AA vs. GG. In a stratified analysis by cancer type, significant association between *Aurora-A* V57I polymorphism and the decreased risk of breast cancer was identified in one genetic model: AA vs. GG. In a stratified analysis by ethnicity, in three genetic models, significant decreased cancer risk was observed among Caucasians (AA vs. GA+GG; AA vs. GG and A vs. G) instead of Asians. Furthermore, a stratified analysis by ethnicity in breast cancer subgroup, five genetic models (AA+GA vs. GG; AA vs. GA+GG; AA vs. GG; AA vs. GA and A vs. G), significant decreased cancer risk was observed among Caucasians, but not among Asians. A slight publication bias was observed in our meta-analysis, thus nonparametric “trim-and-fill” method was utilized to detect the stability of our results. The adjusted odds ratios and confidence intervals showed that *Aurora-A* V57I polymorphism might be a protective factor for cancer risk, suggesting the reliability of our findings.

**Conclusion:**

In summary, this meta-analysis suggests that *Aurora-A* V57I polymorphism may be a protective factor for cancer risk.

## Introduction

Aurora-A protein, also known as STK15/BTAK, belong to the Aurora family of cell cycle-regulating serine/threonine kinase. Aurora-A plays a pivotal role of mitotic centrosome separation, maturation and spindle formation and stability [1], [2]. At the centrosome, Aurora-A is overexpressed in the passage from G2 to M, degraded after termination of cytokinesis, and expressed at significant low levels in G1 and S passage, partly because of effective post-translational degradation through the ubiquitination machinery [3]. Overexpression and altering activity of Aurora-A leads to genomic instability, destroys the accuracy of centrosome duplication, and results in cellular transformation and malignance [4]. The evaluation of the association between single nucleotide polymorphisms (SNPs) in cell cycle regulation genes and cancer risk would be beneficial for further studies. Previous investigations demonstrated that there were two prevalent non-synonymous SNPs (F31I, rs2273535 and V57I, rs1047972) in *Aurora-A* coding region. F31I (rs2273535, T>A) is located on exon 3 of *Aurora-A*, encodes a phenylalanine → isoleucine substitution at amino acid residue 31 [5]. The other SNP, V57I (rs1047972, G>A), also in exon 3 of *Aurora-A*, encodes a valine → isoleucine at amino acid residue 57 [6]. Of late, *Aurora-A* V57I polymorphism was widely investigated for the association between the SNP and cancer risk. A number of investigations indicated that this SNP was a low protective polymorphism in carcinogenesis, particularly in breast cancer [7]. However, the results from these studies remain conflicting rather than conclusive, which may be caused by the insufficient sample sizes, different genotypic milieus and publication bias. To the best of our knowledge, there was no meta-analysis study conducted on the association of *Aurora-A* V57I polymorphism with cancer risk. Thus, we carried out a meta-analysis on overall eligible publications, to confirm whether the V57I polymorphism of *Aurora-A* was associated with cancer risk.

## Materials and Methods

The meta-analysis is reported on the basis of the Preferred Reporting Items for Meta-analyses (PRISMA) guideline (**Table S1**. PRISMA checklist) [8].

### Search Strategy

We searched genetic association articles on PubMed, EMBASE, CBM (Chinese BioMedical Disc) and CNKI (Chinese National Knowledge Infrastructure) databases (published up to June 01, 2013) using combinations of the following terms “Aurora-A”, “BTAK”, “stk15”, “AIKI”, “rs1047972”, “polymorphism”, “SNP”, “mutation”, “locus”, “cancer”, “carcinoma”, “tumor”, “neoplasm” and “malignance”. Additionally, in searching, the language of overall studies was restricted in English or Chinese. The references of retrieved publication, published reviews, letters and comments were scanned for additional relevant studies.

### Inclusion and Exclusion Criteria

For inclusion in this meta-analysis, all eligible articles had to meet the major criteria: 1) focusing on the association of *Aurora-A* V57I polymorphism and cancer risk; 2) case-control or cohort study design; 3) frequencies of genotypes or alleles in case groups and control groups could be exacted from the articles; 4) genotype distributions of controls passed Hardy-Weinberg equilibrium (HWE) test; 5) all cases were diagnosed by pathological examination.

Exclusion criteria were: 1) overlapping data; 2) not case-control studies; 3) only relevant to oncotherapy; 4) review, meta-analysis or letter.

### Data Extraction

In a standardized form, information from all original publications was extracted independently by two reviewers (W. Tang and H. Qiu). In case of disagreements, differences were adjudicated after elaborate discussion among all reviewers. For every eligible article, the following items were extracted: first author's name, cancer type, publication year, country, ethnicity of study subjects, sample size (total cases and controls), genotype method, allele and genotype frequencies and evidence of HWE in controls.

### Statistical analysis

An internet-based HWE test (http://ihg.gsf.de/cgi-bin/hw/hwa1.pl) was used to provide evidences of the HWE in the controls. STATA Version 12.0 software was used to calculate the crude odds ratio (OR) with the corresponding 95% confidence intervals (95% CI) to evaluate the strength of association between *Aurora-A* V57I polymorphism and cancer risk. Z test and *P* value (two-tailed) were used to evaluate the significance of the pooled OR, and if *P*<0.05, statistically significance was confirmed. In current meta-analysis, a Chi-square based I^2^ test was applied to assess the potential heterogeneity among eligible publications, with I^2^ value less than 25% indicating low heterogeneity; 25% to 50% indicating moderate heterogeneity; and greater than 50% indicating high heterogeneity [9]. When I^2^>50% or *P*<0.10, the heterogeneity in publications was considered significant and the random-effects model (the DerSimonian-Laird method) was conducted for meta-analysis [10], otherwise, the fixed-effects model was applied (the Mantel-Haenszel method) [11]. Ethnicity-specific and cancer type-specific effect were evaluated by subgroup analysis (any type of cancer investigated by less than two case-control studies was combined into “other cancers”). The evidence of potential publication bias was evaluated by the funnel plot and Egger's test. Publication bias was assessed by visual inspection an asymmetric plot. Additionally, statistical significance was considered at *P*<0.1 for the interpretation of Egger's test. Sensitivity analyses were conducted to evaluate the stability of the results. Nonparametric “trim-and-fill” method was also utilized to determine the stability of our results. All the statistical manipulations were implemented by using STATA 12.0 statistical software, and all the *P* values were two sided.

## Results

### Characteristics of eligible Studies

**Figure 1:**
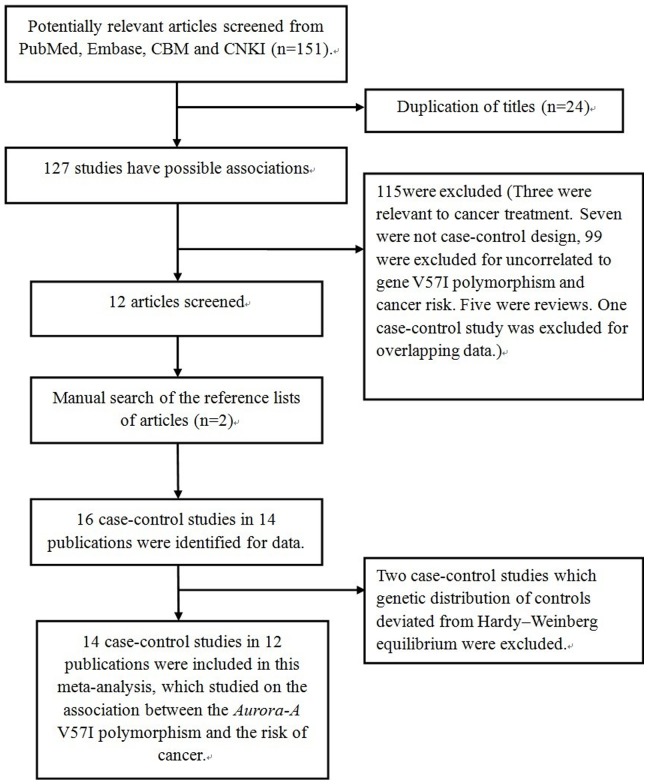
Flow diagram of articles included process for *Aurora-A*V57I polymorphism and cancer risk meta-analysis.

Relevant publications were retrieved from databases (PubMed, Embase, CBM and CNKI). As shown in **Figure** **1**, a total of 151 relevant publications were adopted through reading literatures. Among them, 139 publications were excluded (24 for duplication of titles, seven for non-case-control studies, three for an association with cancer treatment, 99 for not relevant to *Aurora-A* V57I polymorphism and cancer risk, five reviews and one for overlapping data). After this step, 12 papers were identified for data extraction and assessment. After a manual search of the bibliography lists from retrieved, as a result, another two articles were recruited (**Figure 1**). Afterwards, two papers were discarded because the genetic distributions in the control group statistically deviated from HWE [12], [13]. In the study reported by DiCioccio and colleagues [14], there were three independent groups, thus we treated them separately. Lastly, 14 case-control studies [6], [7], [14]–[23] on the association between the *Aurora-A* V57I (rs1047972) polymorphism and cancer risk were recruited in this meta-analysis. Among 14 studies [6], [7], [14]–[23], five investigated breast cancer [6], [7], [15], [16], [21], three investigated ovarian cancer [14], two investigated lung cancer and the others investigated bladder cancer, uterine cancer, gastric cancer and colorectal cancer [14], [17]–[20], [22], [23]. Among these studies, three were from Asians [15]–[17] and 11 from Caucasians [6], [7], [14], [18]–[23]. Characteristics of studies extracted and included in this meta-analysis are exhaustive summarized in **Table 1**, *Aurora-A* V57I genotype and allele frequencies among cancer cases and controls were presented in **Table 2**.

**Table 1:** Characteristics of all included studies in the meta-analysis.

Study	Year	Ethnicity	Country	Cancer type	Sample size (case/control)	Genotype method
						
MARIE-GENICA	2009	Caucasians	German	breast cancer	3139/5469	MALDI-TOF MS
Dogan et al.	2008	Caucasians	Turkish	lung cancer	102/102	Direct sequencing
Ye et al.	2008	Caucasians	USA	bladder cancer	604/593	Taqman
Chen et al.	2007	Caucasians	USA	colorectal cancer	60/65	Direct sequencing
Wang et al.	2007	Caucasians	USA	lung cancer	1263/1154	TaqMan
Milam et al.	2007	Caucasians	USA	uterine cancer	140/188	Taqman
Cox et al.	2006	Caucasians	USA	breast cancer	1240/1724	TaqMan
Ju et al.	2006	Asians	Korea	gastric cancer	501/427	MALDI-TOF MS
Lo et al.	2005	Asians	China(Taiwan)	breast cancer	704/1950	TaqMan
DiCioccio et al.	2004	Caucasians	UK	ovarian Cancer	750/843	TaqMan
DiCioccio et al.	2004	Caucasians	USA	ovarian Cancer	323/427	TaqMan
DiCioccio et al.	2004	Caucasians	Denmark	ovarian Cancer	432/1112	TaqMan
Egan et al.	2004	Caucasians	USA	breast cancer	905/788	Direct sequencing
Dai et al.	2004	Asians	China	breast cancer	1102/1188	TaqMan

MALDI–TOF MS: Matrix-Assisted Laser Desorption/Ionization Time of Flight Mass spectrometry.

**Table 2:** Distribution of *Aurora-A* V57I polymorphism genotype and allele among cases and controls.

	Case			Control			Case		Control		HWE
	AA	GA	GG	AA	GA	GG	A	G	A	G	
MARIE-GENICA	69	850	2220	171	1561	3737	988	5290	1903	9035	Yes
Dogan et al.	5	33	64	5	25	72	43	161	35	169	Yes
Ye et al.	22	162	420	13	148	432	206	1002	174	1012	Yes
Chen et al.	1	20	39	2	20	43	22	98	24	106	Yes
Wang et al.	26	321	916	31	304	819	373	2153	366	1942	Yes
Milam et al.	3	31	106	5	45	138	37	243	55	321	Yes
Cox. et al.	28	342	870	47	462	1215	398	2082	556	2892	Yes
Ju et al.	14	100	387	9	104	314	128	874	122	732	Yes
Lo et al.	15	146	543	30	414	1506	176	1232	474	3426	Yes
DiCioccio et al.(UK)	20	219	511	31	246	566	259	1241	308	1378	Yes
DiCioccio et al.(USA)	9	96	218	14	127	286	114	532	155	699	Yes
DiCioccio et al.(Denmark)	12	109	311	25	282	805	133	731	332	1892	Yes
Egan et al.	23	245	637	21	225	542	291	1519	267	1309	Yes
Dai et al.	16	281	805	17	263	908	313	1891	297	2079	Yes

HWE: Hardy–Weinberg equilibrium.

### Quantitative Synthesis

**Figure 2:**
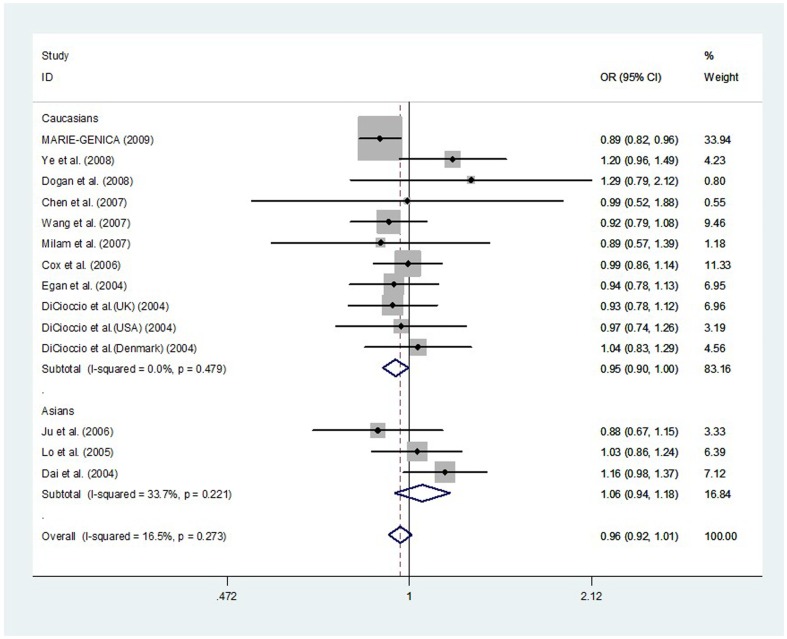
Meta-analysis with a fixed-effects model for the association between the risk of cancer and the *Aurora-A* V57I polymorphism (A vs. G).

In total, our meta-analysis involved 11,245 cancer cases and 16,024 controls from 14 eligible investigations. Our findings demonstrated a borderline association between *Aurora-A* V57I polymorphism and decreased risk of cancer in two genetic models: AA vs. GA+GG (OR, 0.87; 95% CI, 0.74–1.02; *P* = 0.091) and AA vs. GG (OR, 0.87; 95% CI, 0.74–1.02; *P* = 0.077) (**Table 3**). In a stratified analysis by cancer type, we found significant association between *Aurora-A* V57I polymorphism and decreased risk of breast cancer in one genetic model: AA vs. GG (OR, 0.81; 95% CI, 0.66–0.99; *P* = 0.043) (**Table 4**). In a stratified analysis by ethnicity, we found significant decreased cancer risk in three genetic models (OR, 0.82; 95% CI, 0.69–0.98; *P* = 0.025 for AA vs. GA+GG; OR, 0.81; 95% CI, 0.69–0.97; *P* = 0.020 for AA vs. GG and OR, 0.95; 95% CI, 0.90–1.00; *P* = 0.035 for A vs. G) in Caucasians, but not in Asians (**Table 3**, **Figure 2**). Additionally, a stratified analysis by ethnicity was conducted in breast cancer subgroup. We found significant decreased cancer risk in five genetic models (OR, 0.92; 95% CI, 0.86–1.00; *P* = 0.043 for AA+GA vs. GG; OR, 0.75; 95% CI, 0.60–0.94; *P* = 0.014 for AA vs. GA+GG; OR, 0.74; 95% CI, 0.59–0.93; *P* = 0.009 for AA vs. GG; OR, 0.79; 95% CI, 0.62–0.99; *P* = 0.042 for AA vs. GA; and OR, 0.92; 95% CI, 0.86–0.98; *P* = 0.011 for A vs. G) in Caucasians, but not in Asians (**Table 5**).

**Table 3:** Different comparative genetic models results of this meta-analysis in the subgroup analysis by race.

Polymorphism	Genetic comparison	Population	OR(95%CI); *P*	Test of heterogeneity	
				(*p* -Value, I^2^)	Model
	AA+GA vs. GG	All	0.97(0.92–1.02);0.252	0.375,7.0%	F
		Asians	1.02(0.84–1.25);0.839	0.098,57.0%	R
		Caucasians	0.95(0.90–1.01);0.107	0.677,0.0%	F
	AA vs. GA+GG	All	0.87(0.74–1.02);0.091	0.591,0.0%	F
		Asians	1.23(0.82–1.85);0.316	0.783,0.0%	F
		Caucasians	**0.82(0.69**–**0.98);0.025**	0.676,0.0%	F
	AA vs. GG	All	0.87(0.74–1.02);0.077	0.526,0.0%	F
		Asians	1.23(0.82–1.86);0.316	0.853,0.0%	F
		Caucasians	**0.81(0.69**–**0.97);0.020**	0.595,0.0%	F
*Aurora-A* V57I	GA vs. GG	All	0.98(0.93–1.03);0.445	0.519,0.0%	F
		Asians	1.00(0.79–1.25);0.979	0.055,65.6%	R
		Caucasians	0.97(0.91–1.03);0.278	0.859,0.0%	F
	AA vs. GA	All	0.89 (0.75–1.05);0.158	0.730,0.0%	F
		Asians	1.23(0.81–1.87);0.332	0.491,0.0%	F
		Caucasians	0.84(0.70–1.00);0.054	0.862,0.0%	F
	A vs. G	All	0.96 (0.92–1.01);0.128	0.273,16.5%	F
		Asians	1.06(0.94–1.18);0.351	0.221,33.7%	F
		Caucasians	**0.95(0.90**–**1.00);0.035**	0.479,0.0%	F

F indicates fixed model; R indicates random model.

**Table 4:** Different comparative genetic models results of this meta-analysis in the subgroup analysis by cancer type.

Polymorphism	Genetic comparison	Cancer type	OR(95%CI); *P*	Test of heterogeneity	
				(*p* -Value, I^2^)	Model
	AA+GA vs. GG	All	0.97(0.92–1.02);0.252	0.375,7.0%	F
		Breast cancer	0.99(0.89–1.10);0.841	0.085,51.1%	R
		Ovarian Cancer	0.98(0.85–1.13);0.792	0.925,0.0%	F
		Lung cancer	0.96(0.81–1.14);0.642	0.167,47.6%	F
		Other cancers	1.00(0.84–1.19);0.991	0.313,15.7%	F
	AA vs. GA+GG	All	0.87(0.74–1.02);0.091	0.591,0.0%	F
		Breast cancer	0.82(0.67–1.00);0.054	0.326,13.9%	F
		Ovarian Cancer	0.88(0.59–1.30);0.518	0.487,0.0%	F
		Lung cancer	0.79(0.49–1.29);0.349	0.698,0.0%	F
		Other cancers	1.36(0.84–2.21); 0.216	0.693,0.0%	F
	AA vs. GG	All	0.87(0.74–1.02);0.077	0.526,0.0%	F
		Breast cancer	**0.81(0.66–0.99);0.043**	0.277,21.6%	F
		Ovarian Cancer	0.88 (0.59–1.30);0.514	0.485,0.0%	F
		Lung cancer	0.80 (0.49–1.30);0.359	0.567,0.0%	F
		Other cancers	1.35(0.83–2.21);0.225	0.661,0.0%	F
*Aurora-A* V57I	GA vs. GG	All	0.98(0.93–1.03);0.445	0.519,0.0%	F
		Breast cancer	0.98(0.91–1.05);0.508	0.145,41.5%	F
		Ovarian Cancer	0.99(0.86–1.15);0.915	0.996,0.0%	F
		Lung cancer	0.98(0.82–1.17);0.816	0.169,47.1%	F
		Other cancers	0.97(0.81–1.16);0.717	0.344,9.7%	F
	AA vs.GA	All	0.89 (0.75–1.05);0.158	0.730,0.0%	F
		Breast cancer	0.84(0.68–1.04);0.103	0.465,0.0%	F
		Ovarian Cancer	0.88(0.59–1.33);0.552	0.524,0.0%	F
		Lung cancer	0.79(0.48–1.31);0.357	0.949,0.0%	F
		Other cancers	1.40(0.84–2.31);0.197	0.748,0.0%	F
	A vs. G	All	0.96 (0.92–1.01);0.128	0.273,16.5%	F
		Breast cancer	0.98(0.89–1.08);0.749	0.062,55.4%	R
		Ovarian Cancer	0.97(0.86–1.10);0.663	0.770,0.0%	F
		Lung cancer	0.95 (0.82–1.10);0.485	0.202,38.6%	F
		Other cancers	1.03(0.88–1.20);0.699	0.313,15.7%	F

F indicates fixed model; R indicates random model.

**Table 5:** Different comparative genetic models results of this meta-analysis in the breast cancer subgroup analysis by ethnicity.

Polymorphism	Genetic comparison	Population	OR(95%CI); *P*	Test of heterogeneity	
				(*p* -Value, I^2^)	Model
	AA+GA vs. GG	All	0.99(0.89–1.10);0.841	0.085,51.1%	R
		Asians	1.10(0.96–1.27);0.158	0.222,32.9%	F
		Caucasians	**0.92(0.86–1.00);0.043**	0.403,0.0%	F
	AA vs. GA+GG	All	0.82(0.67–1.00);0.054	0.326,13.9%	F
		Asians	1.20(0.75–1.91);0.441	0.504,0.0%	F
		Caucasians	**0.75(0.60–0.94);0.014**	0.600,0.0%	F
	AA vs. GG	All	**0.81(0.66–0.99);0.043**	0.277,21.6%	F
		Asians	1.22(0.77–1.95);0.396	0.574,0.0%	F
		Caucasians	**0.74(0.59–0.93);0.009**	0.565,0.0%	F
*Aurora-A* V57I	GA vs. GG	All	0.98(0.91–1.05);0.508	0.145,41.5%	F
		Asians	1.10(0.95–1.26);0.205	0.154,50.7%	F
		Caucasians	0.94(0.87–1.02);0.147	0.463,0.0%	F
	AA vs.GA	All	0.84(0.68–1.04);0.103	0.465,0.0%	F
		Asians	1.13(0.70–1.83);0.614	0.329,0.0%	F
		Caucasians	**0.79(0.62–0.99); 0.042**	0.679,0.0%	F
	A vs. G	All	0.98 (0.89–1.08);0.749	0.062,55.4%	R
		Asians	1.10(0.97–1.25);0.139	0.369,0.0%	F
		Caucasians	**0.92(0.86–0.98);0.011**	0.376,0.0%	F

F indicates fixed model; R indicates random model.

### Tests for Publication Bias, Sensitivity Analyses, and Heterogeneity

**Figure 3:**
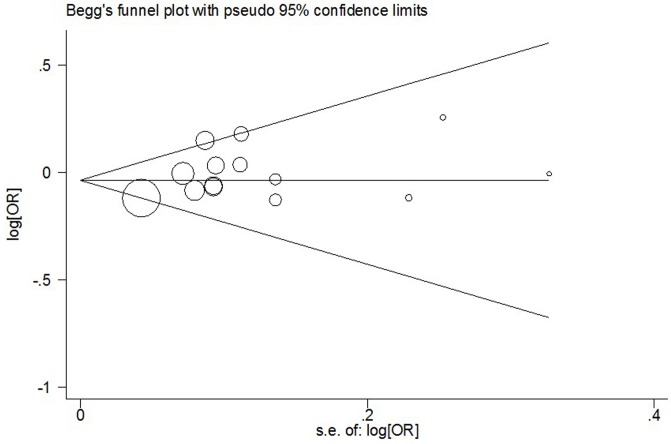
For *Aurora-A* V57I polymorphism, Begg's funnel plot analysis for publication bias for overall cancer in the dominant model.

Begg's funnel plot and Egger's test were carried out to measure publication bias. The shape of funnel plot revealed the evidence of funnel plot symmetry (**Figure 3**). The statistical results indicated that there was a slight publication bias in this meta-analysis (A vs. G: Begg's test *P* = 0.511, Egger's test *P* = 0.113; AA vs. GG: Begg's test *P* = 0.443, Egger's test *P* = 0.083; GA vs. GG: Begg's test *P* = 0.324, Egger's test *P* = 0.271; AA vs. GA: Begg's test *P* = 0.324, Egger's test *P* = 0.190; dominant model: Begg's test *P* = 0.584, Egger's test *P* = 0.170; recessive model: Begg's test *P* = 0.511, Egger's test *P* = 0.103).

**Figure 4:**
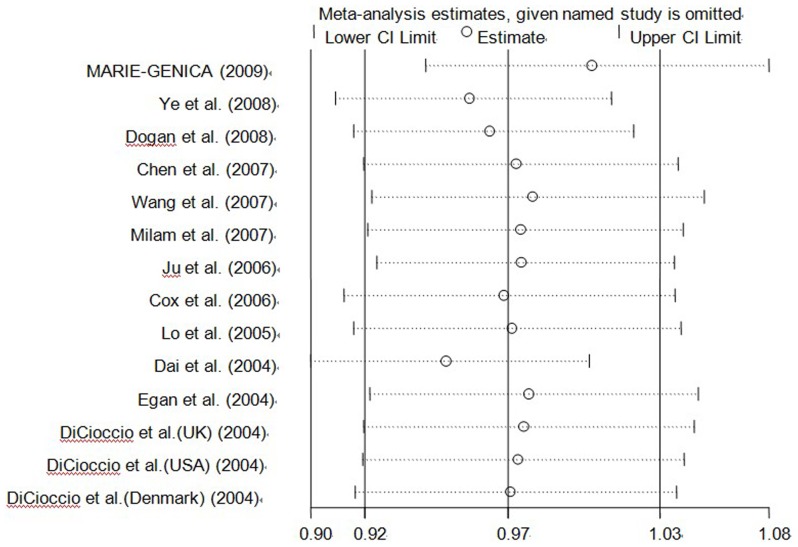
Sensitivity analysis of the influence of dominant model in overall cancer meta–analysis (fixed–effects estimates).

**Figure 5:**
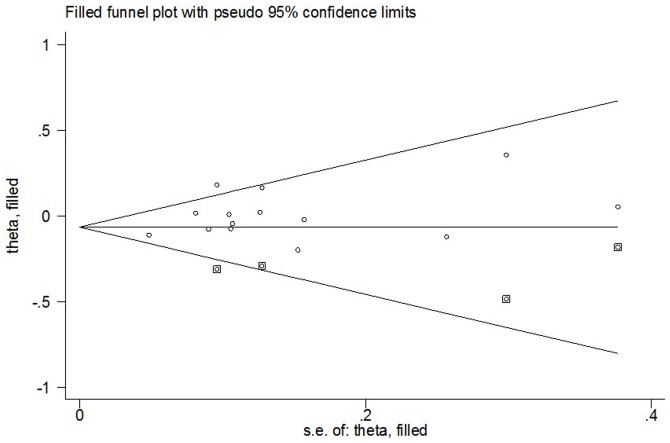
Filled funnel plot of meta-analysis of between the *Aurora-A*V57I polymorphism and EC risk (AA+GA vs. GG).

An individual study involved in the meta-analysis was omitted in turn, to confirm the influence of individual dataset to the pooled ORs, and the statistical significances of all genetic comparison models were not qualitatively altered (**Figure 4**) (data not shown). Nonparametric “trim-and-fill” method was utilized as the other sensitivity analysis method. The adjusted ORs and CIs showed that *Aurora-A* V57I polymorphism may be a protective factor for cancer risk (A vs. G: adjusted pooled OR = 0.92, 95% CI: 0.88–0.96, *P* = 0.000; AA vs. GG: adjusted pooled OR = 0.76, 95% CI: 0.66–0.88, *P* = 0.000; dominant model: adjusted pooled OR = 0.94, 95% CI: 0.89–0.98, *P* = 0.010; recessive model: adjusted pooled OR = 0.76, 95% CI: 0.66–0.88, *P* = 0.000; GA vs. GG: adjusted pooled OR = 0.95, 95% CI: 0.90–1.00, *P* = 0.041; AA vs. GA: adjusted pooled OR = 0.89, 95% CI: 0.76–1.05, *P* = 0.174) (**Figure 5**).

The results showed there were relatively low heterogeneities among the recruited studies. Since tumor origin and ethnicity might influence the results of meta–analysis, we implemented subgroup analyses by cancer type and ethnicity (**Table 3** **and** **Table 4**). The results indicated that breast cancer, lung cancer, Asian populations might contribute to the heterogeneity.

## Discussion

Recently, many studies have indicated that genovariation plays a crucial role in individual susceptibility to complex disease, such as cancer and autoimmune disease [24], [25]. Functional polymorphisms, which impact on the regulation of gene expression, can lead to differences between individuals in the risk to multiple cancers [26]. Early studies have demonstrated that *Aurora-A* is an oncogene situated on chromosome 20q13.2, a region constantly amplified in a number of human cancers [27]–[29]. Recent investigations have showed that regulated *Aurora-A* is pivotal for maintenance of chromosome integrity after DNA damage, and *Aurora-A* polymorphisms affect some important functions of Aurora-A protein [30]. Some investigations have demonstrated that *Aurora-A* gene SNPs may associate with the production of the Aurora-A protein on the process of carcinogenesis [31], [32].

In decade, several molecular epidemiological studies have been performed to assess the association of *Aurora-A* V57I (rs1047972 G>A) polymorphism with cancer risk. However, the results were conflicting. Thus, we conducted a comprehensive meta-analysis involving published data, to assess the strength of association between *Aurora-A* V57I polymorphism and cancer risk. In current meta-analysis, a total of independent 14 case-control studies in 12 publications including 11,245 cases and 16,024 controls were recruited, and then identified the association of *Aurora-A* V57I polymorphism with cancer risk. Our results demonstrated a borderline association between the *Aurora-A* V57I polymorphism and decreased risk of cancer in two genetic models: AA vs. GA+GG and AA vs. GG. In a stratified analysis by cancer type, our results indicated that *Aurora-A* V57I polymorphism was associated with a significantly decreased risk of breast cancer in AA vs. GG genetic model. In a stratified analysis by ethnicity, a protective effect of *Aurora-A* V57I polymorphism was observed in Caucasians, but not in Asians. In addition, in a stratified analysis by ethnicity in breast cancer subgroup, five genetic models significant decreased cancer risk were observed among Caucasians (AA+GA vs. GG; AA vs. GA+GG; AA vs. GG; AA vs. GA and A vs. G), but not among Asians.

It has been observed that the *Aurora-A* F31I polymorphism, converts the activity of the Aurora-A box-1, leading to obstruction of p53 binding and reducing the degradation of Aurora-A [1]. The stable overexpression of Aurora-A results in centrosome amplification, chromosomal instability and promotion of tumorigenesis [1]. *Aurora-A* V57I polymorphism, which results in a valine (val) to isoleucin (Ile) substitution at codon 57, is associated with subdued celluar transformation and increased chromosomal stability. The *Aurora-A* V57I polymorphism in *Aurora-A* box-2 does not regulate Aurora-A degradation, but may affect the secondary structure and function of *Aurora-A* F31I polymorphism [6]. Our results demonstrated that the V57I mutation in *Aurora-A* decreased the risk of cancer, perhaps by modifying the function of *Aurora-A* F31I polymorphism and altering the secondary structure of protein.

Since cancer origins might affect the outcomes from meta-analysis, we performed a stratified analysis by cancer type for *Aurora-A* V57I polymorphism. The results indicated that *Aurora-A* V57I polymorphism was associated with a decreased risk of breast cancer, but not of ovarian cancer, lung cancer and other cancers. However, our results should be interpreted with very caution. Only five case-control studies for breast cancer, three for ovarian cancer and two for lung cancer were included in this meta-analysis, which might reduce statistical power to get a reliable result. Future, more large-scale studies should be conducted to confirm or refute these results. Because ethnicity could also affect the results of meta-analysis, we performed a stratified analysis by race for *Aurora-A* V57I polymorphism. Our results demonstrated the *Aurora-A* V57I polymorphism was a protective factor in Caucasians but not in Asians. In current meta-analysis, only three investigations of Asians were obtained. The outcomes might be due to fluke because the limited number of eligible studies and sample sizes might lead to deficient statistical power to measure a minor effect. Thus, our results should also be interpreted with very caution. In the future, further studies with large sample sizes regarding Asians should be carried out to identify the possible effects of *Aurora-A* V57I polymorphism ethnic variations on cancer risk.

Two significant issues, heterogeneity and publication bias, should be addressed. A slight publication bias and relatively low heterogeneity were observed in our meta-analysis. As subgroup analyses were performed according to ethnicity and cancer type, Asian populations, lung cancer and breast cancer subgroups contributed the major source of heterogeneity. Since publication bias was observed, nonparametric “trim-and-fill” method was utilized to detect the stability of our results. The adjusted ORs and CIs show that *Aurora-A* V57I polymorphism may be a protective factor for cancer risk, suggesting the reliability of our findings.

Although our results were stable and suggestive, there were several limitations in this study which should be acknowledged. First, only 14 eligible case-control studies in 12 publications were recruited in this meta-analysis; therefore, the results might be a fluke, as the limited number and sample sizes of eligible studies could lead to deficient statistical power to detect a real influence. Second, all eligible case-control studies were from Asians and Caucasians; thus, our results only proper for these two populations. Third, a slight publication bias was observed in our meta-analysis. Only twelve eligible publications were recruited in current meta-analysis, so some unpublished studies were unavoidably missed, which may result in bias. Fourth, high heterogeneity existed in some subgroups. This could be due to other differences between publications, such as age, gender, cancer type, ethnicity, smoking, drinking, other lifestyle factors, environmental risk factors and unscreened controls as well. For limited number of eligible studies or lack of sufficient information in a uniform criterion in investigations, these factors were not considered. Fifth, the *Aurora-A* V57I and F31I polymorphism locate on the same *Aurora-A* exon and significant linkage disequilibrium was found between the two coding SNPs, given the V57I polymorphism might play an important role in the process of carcinogenesis though altering the secondary structure and function of *Aurora-A* F31I polymorphism, thus, this important SNP, *Aurora-A* F31I, should not be ignored. Sixth, in this meta-analysis, some cancers only had one study included; therefore, any type of cancer investigated by less than two case-control studies was combined into “other cancers”, which might lead to heterogeneity in this subgroup.

In conclusion, this meta-analysis indicates that the *Aurora-A* V57I polymorphism may be a protective factor for cancer risk, especially, in Caucasians and breast cancer. As only 14 studies were recruited in this meta-analysis and current evidence was limited, in the future, more large-scale studies with an adequate methodological quality and properly controlling should be carried out, to confirm or refute the relationship between *Aurora-A* V57I polymorphism and the risk of cancer.

## Supporting Information

Checklist S1 **PRISMA checklist, Checklist of items to include when reporting a systematic review or meta-analysis (diagnostic review consisting of cohort studies).**
(DOCX)
